# Residual Ductility and Microstructural Evolution in Continuous-Bending-under-Tension of AA-6022-T4

**DOI:** 10.3390/ma9030130

**Published:** 2016-02-26

**Authors:** Milovan Zecevic, Timothy J. Roemer, Marko Knezevic, Yannis P. Korkolis, Brad L. Kinsey

**Affiliations:** Department of Mechanical Engineering, University of New Hampshire, Durham, NH 03824, USA; mz2@unh.edu (M.Z.); tim@roemer.net (T.J.R.); marko.knezevic@unh.edu (M.K.); Yannis.Korkolis@unh.edu (Y.P.K.)

**Keywords:** continuous-bending-under-tension, aluminum alloys, ductility, anisotropy, crystallographic texture, voids

## Abstract

A ubiquitous experiment to characterize the formability of sheet metal is the simple tension test. Past research has shown that if the material is repeatedly bent and unbent during this test (*i.e.*, Continuous-Bending-under-Tension, CBT), the percent elongation at failure can significantly increase. In this paper, this phenomenon is evaluated in detail for AA-6022-T4 sheets using a custom-built CBT device. In particular, the residual ductility of specimens that are subjected to CBT processing is investigated. This is achieved by subjecting a specimen to CBT processing and then creating subsize tensile test and microstructural samples from the specimens after varying numbers of CBT cycles. Interestingly, the engineering stress initially increases after CBT processing to a certain number of cycles, but then decreases with less elongation achieved for increasing numbers of CBT cycles. Additionally, a detailed microstructure and texture characterization are performed using standard scanning electron microscopy and electron backscattered diffraction imaging. The results show that the material under CBT preserves high integrity to large plastic strains due to a uniform distribution of damage formation and evolution in the material. The ability to delay ductile fracture during the CBT process to large plastic strains, results in formation of a strong <111> fiber texture throughout the material.

## 1. Introduction

In the United States, federal regulations on vehicle fuel economy stipulate that the CAFE (Corporate Average Fuel Economy) standard will be increased from 30.2 mpg in 2011 to 41 mpg by 2016 and to as high as 61 mpg by 2025 [1]. In addition to switching to smaller vehicles, one means to achieve these stringent demands is by significantly reducing the weight of the vehicle body, which accounts for two-thirds of the total weight. Simultaneously, customers demand lower cost and increased comfort and safety for their vehicles. To achieve these lighter, higher performing and more crashworthy structures, new Advanced High Strength Steels (AHSS) such as DP780 and Aluminum alloys such as AA-6022-T4 are incorporated in designs. However, all of these advanced alloys suffer from limited ductility, e.g., less than 10%–20% [2,3,4,5], during sheet metal forming operations. This fuels the research on innovative forming processes such as Continuous-Bending-under-Tension (CBT), which achieves strain levels well above those generally observed in conventional forming.

**Figure 1:**
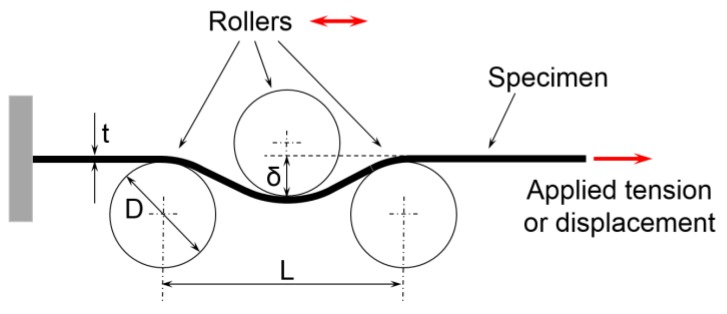
Schematic of the continuous-bending-under-tension (CBT) loading.

As shown by [6], plastic bending-under-tension lowers the axial force that is required for a given deformation of the material, in comparison to pure stretching. In the CBT test, a strip of the material is stretched in tension, while at the same time an assembly of three rollers arranged as in Figure 1 reciprocates along the gage length. As a result, in CBT the material is deformed mainly when subjected to the 3-point bending, with the axial tensile force being used to prevent slack in the specimen and to facilitate the plastic flow over the rollers. This suppresses the usual localization of deformation into a necking region (which limits the uniform elongation in the standard tension test) and allows the material to be extended further without failure. Ultimately, the material fails at multiple necking locations, indicating that the ductility has been depleted uniformly throughout the gauge length.

Benedyk *et al.* [7] were the first to propose using the CBT process to improve the elongation-to-fracture in both ferrous and non-ferrous materials. Scanning electron fractographs of mild steel samples were included showing little visible difference between tensile and CBT fracture faces. Emmens and van den Boogaard [8] further investigated the CBT phenomenon by evaluating the process parameter space. Pulling speed and depth were determined to have the most significant effect on the total elongation achievable. In addition, further research [9] investigated a simple mechanics-based model for the CBT effect and showed that the phenomenon was more dependent on process parameters than the material used. Finally, Emmens and van den Boogaard [10] investigated the CBT process through secondary tensile tests after CBT processing and interrupted CBT tests. This allowed the extension of the stress-strain curve to large strain values. In other research, Nikhare *et al.* [11] performed a numerical investigation of CBT using non-linear kinematic hardening and found that the material model parameters are critical to accurately predict the CBT response. Compressive stresses in the cross-section during CBT were not numerically predicted, which prompted the current, more expansive and detailed investigation of the process.

In this paper, results from microstructural evaluations and residual ductility measurements after CBT processing of specimens are presented. Scanning Electron Microscopy (SEM) images of samples subjected to CBT and subsequent simple tension show that ductile damage develops much more uniformly throughout the material during CBT than during simple tension. Electron Backscatter Diffraction (EBSD) images and corresponding pole figures showing crystallographic texture in the material indicate the specimens subjected to CBT processing develop a strong <111> fiber texture with the number of cycles. The <111> fiber is known to develop during simple tension of face-centered cubic (FCC) ductile metals and is strong only in the necked region. However, with increasing number of CBT cycles a strong <111> fiber develops uniformly throughout the sheet. Furthermore, the residual ductility of the material was assessed by creating subsize tensile test samples from specimens that were subjected to varying numbers of CBT cycles. The results provide further knowledge related to the improved ductility observed during CBT processing of materials.

## 2. Microstructure and Tensile Properties of AA-6022-T4

**Figure 2:**
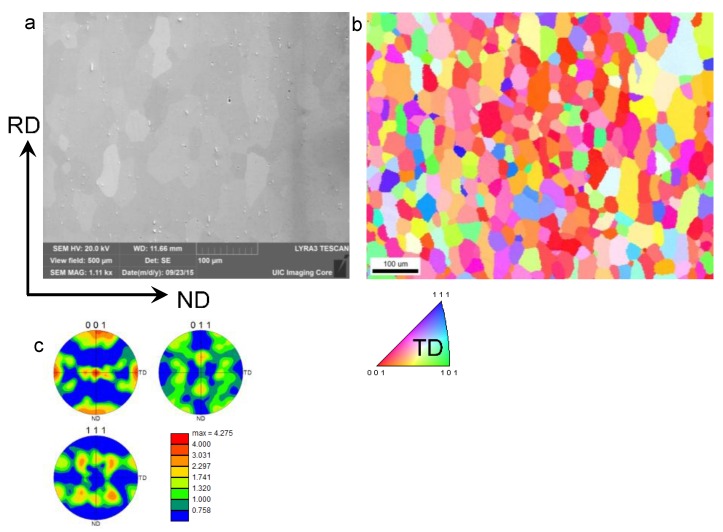
Microstructure and texture of as received AA6022-T4: (**a**) Secondary electron micrograph showing particles and hydrogen pores; (**b**) Electron backscatter diffraction (EBSD) orientation map showing grain structure; and (**c**) pole figures showing crystallographic texture.

The experiments described in this paper were performed on 1 mm thick cold-rolled aluminum AA-6022-T4 sheets. AA-6022 is an Al-Si-Mg alloy, the exact chemical composition of which is given in Table 1. The material was in a temper T4 condition. SEM and EBSD orientation imaging were used to characterize the initial microstructure in terms of voids, particles, grain size, morphology and crystallographic texture. For this imaging, the material was mechanically prepared using automated grinding and polishing procedures. For grinding, 320, 400, 600, 800, and 1200 SiC papers were used. After grinding the samples were polished using a sequence of oil-based diamond suspensions of 6, 3, and 1 μm, respectively. Fine-polishing of the samples was achieved with 0.06 µm colloidal silica suspension. The automated EBSD data collection was performed using the Pegasus system (Octane Plus SDD detector and Hikari High Speed Camera) attached to a Tescan Lyra (Ga) field emission SEM at a voltage of 20 kV. The EBSD scans were run with either 1.5 or 2 μm step size. Figure 2a shows the SEM image of the initial microstructure. Consistent with the literature [12], particles and hydrogen micropores can be observed. Figure 2b,c shows the microstructure and texture of the as-received material. The average grain size in the material is approximately 50 μm with grains slightly elongated in-plane (*i.e.*, in the rolling direction (RD) and the transverse direction (TD)) relative to the normal direction (ND). Pole figures show a typical cube texture component in the material, as a consequence of rolling and annealing.

**Figure 3:**
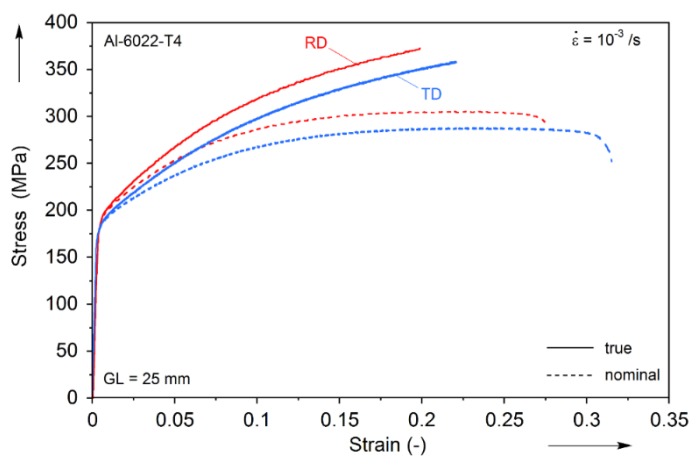
Simple tension stress-strain curves for AA-6022-T4 in the rolling and transverse directions (RD and TD).

The plastic flow of the material along the rolling and transverse directions (RD and TD, respectively) was measured by uniaxial tension tests performed on specimens prepared according to the ASTM E-8 standard. The experiments were performed on a MTS Landmark 370 (Eden Prairie, MN, USA) servohydraulic testing machine with a Flextest 40 controller (Eden Prairie, MN, USA), the MTS 793 data acquisition and control software and the MTS 647 hydraulic wedge grips. The experiments were performed under a nominal strain-rate of 10^−3^/s in the test-section. During testing, both an MTS 634.12E-24 extensometer and a 2D Digital Image Correlation system (VIC-2D from Correlated Solutions, Inc., Columbia, SC, USA) were used to acquire the strain, as well as the strain fields and the plastic-strain ratios (R-values, not reported here). The stress-strain results are shown in Figure 3, and reveal that this material exhibits some mild anisotropy between the RD and TD. This anisotropy can be associated to a preferred distribution of crystal orientations (Figure 2c) and accompanying preferential growth of precipitates along the crystallographic <001> direction [13], which are a consequence of prior processing.

## 3. Continuous-Bending-under-Tension Experiments

### 3.1. Custom CBT Testing Equipment

**Figure 4:**
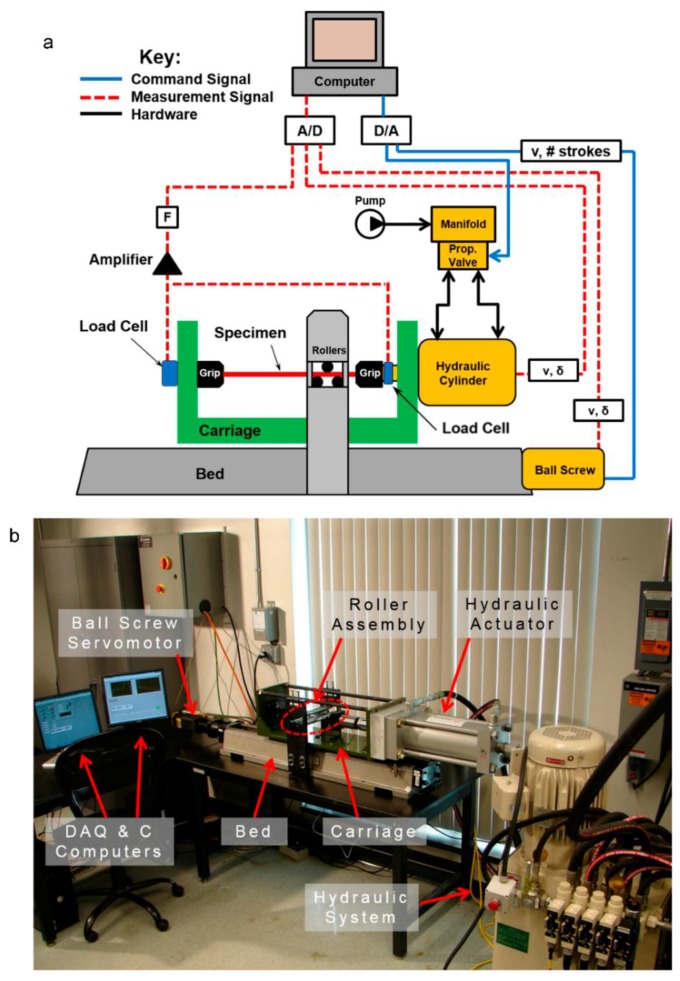
CBT machine at the University of New Hampshire: (**a**) Schematic and (**b**) photograph, with the main components identified.

The CBT experiments were performed on a testing machine custom-built for this work. This machine differs from earlier ones [7,8] in that the three rollers are stationary (non-translating but free to rotate) and the material undergoes CBT flows through them. While not reported here, this arrangement allows for a direct observation, in real-time, of the process zone around the three rollers. Furthermore, the machine is designed for CBT testing of wide sheets, beyond the strips reported here. The custom machine consists of four subsystems: (1) a moving carriage, that contains the specimen, the grips, two load cells (one on each grip) and a hydraulic actuator; (2) a stationary assembly of three rollers; (3) the base (aka bed), which includes an AC brushless servomotor and a ball-screw connected to the carriage which is used for moving it; and (4) the data-acquisition and control system. A schematic of the custom CBT machine is given in Figure 4a, and a photograph, with the main components identified, is provided in Figure 4b.

The box-shaped moving carriage contains a hydraulic actuator rated for 310 kN with a stroke of 300 mm. The actuator has a Micropulse BTL7-A501-M0305-Z-S32 position sensor (Balluff, Neuhausen, Germany) with a resolution of 5 µm and is connected to an 11.2 kW high-pressure hydraulic pump of 210 bar and 32 L/min capacity. This allows a maximum velocity of 33.5 mm/s for tension of the specimen. Two donut-style load-cells, one tension (LCF450, capacity of 22.24 kN, Futek, Irvin, CA, USA) and the other compression (LTH500, capacity of 22.24 kN, Futek, Irvin, CA, USA) are attached to the actuator and the carriage, respectively. The specimen is held between two wedge-type grips.

The stationary roller-assembly contains three stainless-steel rollers of 25 mm diameter which are supported on self-aligning, spherical sliding bearings. For the 1 mm-thick sheet used here, the rollers induce a maximum bending strain of 4%. The axes of the two lower rollers are 54 mm apart. The vertical position of the top roller is adjustable, so that the bending depth δ (see Figure 1) can be set, as well.

The moving carriage and the roller-assembly are connected to a cast-iron bed, which also houses a 1 kW AC brushless servomotor and a ball-screw. The nut of the ball-screw is connected to the bottom of the carriage, setting the latter to a reciprocating motion during the CBT testing. The maximum velocity of the carriage is 66 mm/s.

Finally, a custom program was created in LabVIEW (Austin, TX, USA) for data-acquisition and control. The system schematic is given in Figure 4a. Not shown in that schematic are two limit-switches, one mounted on the moving grip and the other at the bed, which control the limits of the carriage motion. Every time one of these switches is tripped (*i.e.*, when the grips are a specified distance close to the rollers), the carriage is commanded to change direction. Thus, the carriage stroke increases as the specimen elongates during CBT. This is a unique feature of this custom-built machine compared to ones in the literature. The carriage decelerates, momentarily dwells, and accelerates when changing directions.

### 3.2. Representative CBT Experiment

**Figure 5:**
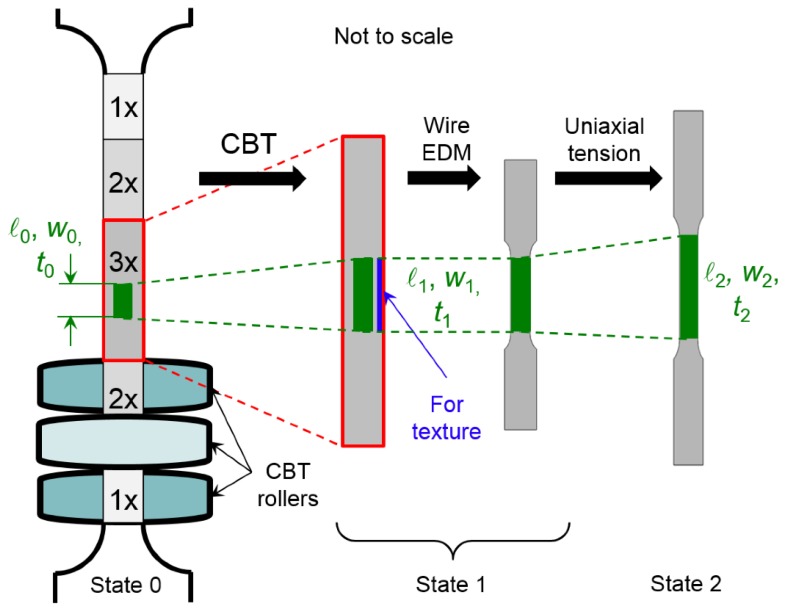
Schematic illustrating the interrupted CBT experiments.

A special geometry was devised for the CBT specimens, using the ASTM E-8 standard geometry as a reference. The specimen consists of a reduced-width section (11.7 mm) and two wider (19.5 mm) shoulders used for gripping, with R12.7 mm fillets between them. The reduced-width section consists of a central region, which is visited by all three rollers during each CBT stroke, and, on either side, two regions that are visited by two rollers only, and two further out regions that are visited by only one roller. These regions are identified as 3×, 2×, and 1×, respectively in Figure 5. The length of the central, 3× region is set to 50 mm, while the lengths of the remaining four regions are determined by the distance between the rollers with the stipulation that the rollers never leave the reduced-width section during testing. As a result, the initial length of the reduced-width section is calculated to be 190 mm.

In a CBT experiment, the specimen is initially placed in the machine and held between the grips. Subsequently, the top roller is lowered to the appropriate depth δ, bending the specimen and developing some axial tension. This tension is necessary to ensure that there is no slack in the specimen before the CBT experiment begins. During testing, the loads on either sides of the CBT specimen and the actuator displacement are recorded by the load-cells and the Micropulse position sensor, respectively. A stroke is defined as the material passing through the rollers and a cycle as two strokes, with the carriage moving down and back.

**Figure 6:**
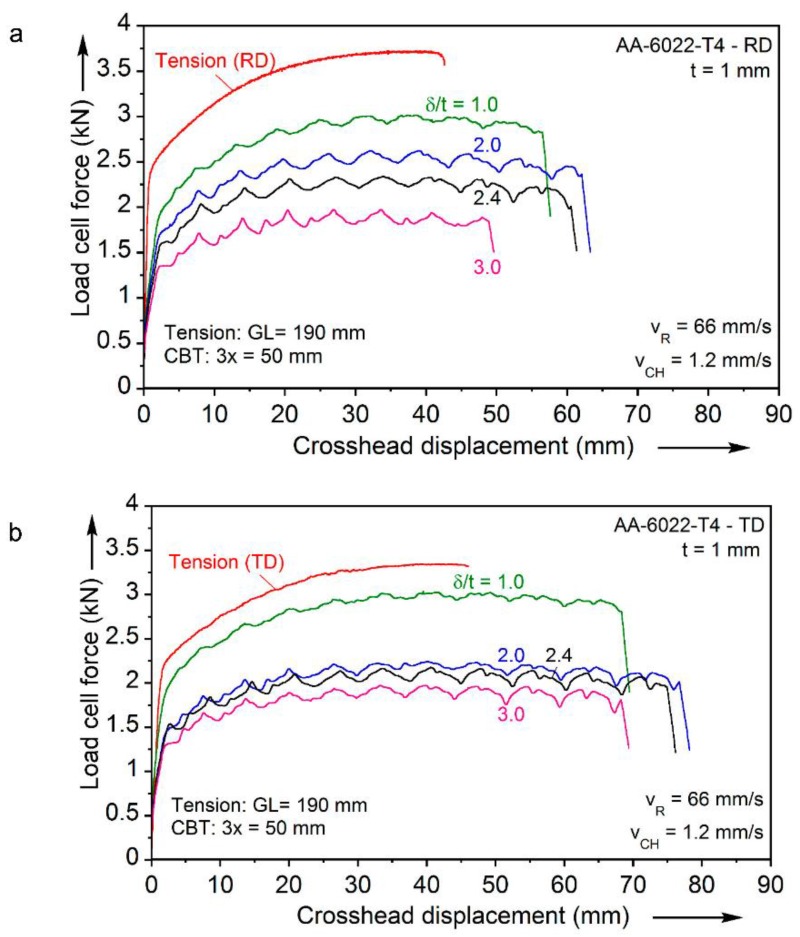
Load-displacement curves for the simple tension and CBT loadings in: (**a**) the RD and (**b**) the TD.

The parameters that can be varied between experiments are the bending depth (normalized by the initial specimen thickness) δ*/t*_0_, the hydraulic cylinder tension (or crosshead) velocity *V_CH_* and the carriage (or roller) velocity *V_R_*. An initial investigation of this parameter space in terms of the maximum elongation-to-fracture achievable was reported by Roemer *et al.* [14]. Some of these results are presented in Figure 6a,b, in the form of axial load-end displacement results recorded during the CBT tests. In each plot, the response of a tension test is included. The experiment is performed on a CBT specimen, *i.e.*, with a reduced-section of 190 mm long, which is uniformly stretched in tension until localization sets-in. In contrast, in the CBT tests only 50 mm of the test-section, *i.e.*, the 3× region, are visited by all three rollers and receive the full number of bending-unbending cycles. Therefore, the displacements shown underrepresent the increased displacements in the CBT tests compared to those of the tension tests. Therefore, the overall elongation-to-fracture under the CBT conditions is in every case significantly greater than the one obtained under conventional tension.

In almost every CBT experiment, the forces recorded are below the yield limit of the material. This is in agreement with earlier CBT work [8,9,10], where it was postulated that the material deforms plastically only when it is bent and unbent by the rollers. This deformation mode explains the suppression of the usual localization of deformation that occurs in the conventional tension test. Macroscopically, this suppression is perceived as an enhanced elongation-to-fracture under CBT loading. One of the objectives of this paper is to explain, at least qualitatively, this observation in terms of the material microstructure evolution.

The force-displacement curves exhibit a periodic waviness, as in the earlier works by Emmens and van den Boogaard [8,9,10], which correspond to the end of a stroke and the reversal of the carriage movement. A more detailed explanation of these shapes is in [15], with additional analyses, using the synchronous data from both load-cells.

Finally, it is remarkable that the failure of the AA-6022-T4 material exhibits significant anisotropy, with the TD having a significantly larger elongation-to-fracture in comparison to the RD (see Figure 5). This is in agreement with the anisotropy in failure recorded during conventional tension (see Figure 3). Values related to this are RD to TD difference of 22% and 40% for the uniaxial tension and CBT, with a δ*/t*_0_ = 3 depth, cases respectively.

### 3.3. Interrupted CBT Experiments

Motivated by the earlier work by Emmens and van den Boogaard [10], the CBT experiment was used to probe the hardening response of the material past the limit of uniform deformation in uniaxial tension. At the same time, a detailed study of the microstructural evolution during the CBT loading was performed, as described in the next section.

For these “interrupted CBT” experiments, the procedure depicted in Figure 5 was used. First, a batch of specimens was loaded with varying number of CBT cycles, as described in Table 2. (A CBT cycle is defined as the rollers traversing the entire gauge length and then back again.) Two families of specimens were tested, one in the RD and the other in TD. In every case, the central region was visited by all three rollers during each stroke (*i.e.*, the 3× region), and which therefore was loaded to the number of CBT cycles denoted in Table 2, and marked before the CBT experiments (see Figure 5). After the specified number of cycles, the specimen was removed from the CBT machine. Depending on the number of CBT cycles, the specimens were found to have an increasing amount of residual curvature when removed from the CBT machine. A subsize tensile specimen (ASTM E-8) was then extracted from the marked central region (3×) of the parent CBT specimen using wire-EDM. In every case, the test-section of the subsize tensile specimen was well inside the 3× region of the parent CBT specimen, as shown in the schematic of Figure 5. At the same time, the material on the side of the gauge section (approximate 1 × 2 × 50 mm^3^ in size but different for each specimen due to varying number of CBT cycles), which was removed during wire-EDM processing of the subsize tensile specimens, was saved for subsequent microstructural studies (see next section). Finally, the subsize tensile specimens were loaded in uniaxial tension using the equipment of Section 3.1.

**Figure 7:**
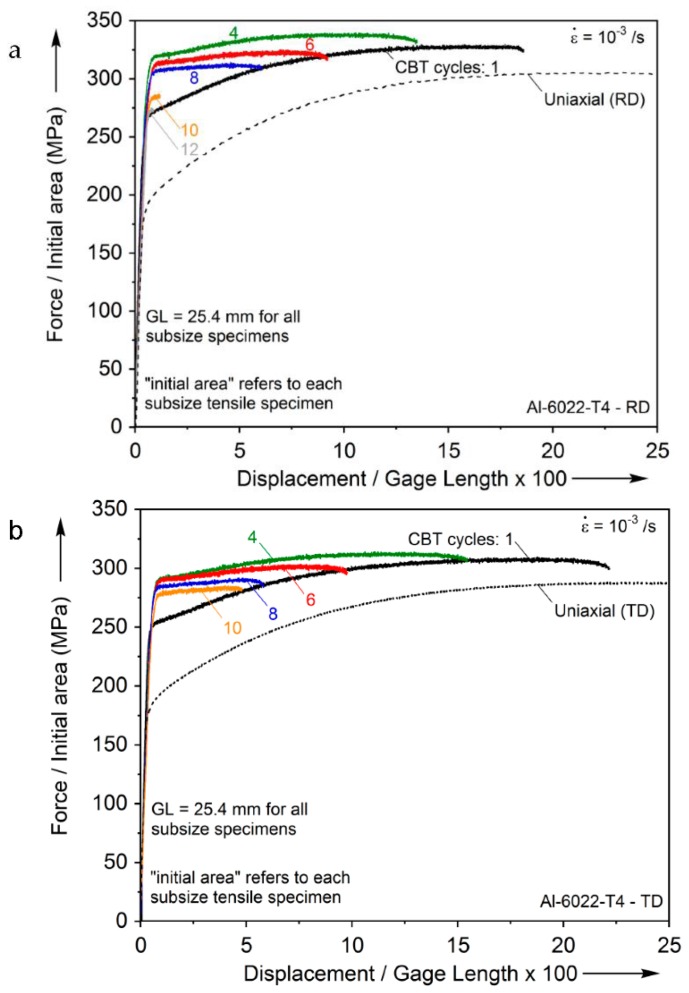
Engineering stress-normalized extension curves from the interrupted CBT experiments in: (**a**) the RD and (**b**) the TD.

The engineering stress-strain curves from these subsequent tension tests are plotted in Figure 7 along with the uniaxial tension tests of the as-received materials (see Figure 3). It can be seen that the load-carrying capacity of the subsize specimens has increased over the as-received one, while their residual elongation-to-fracture is reduced. Furthermore, the load-carrying capacity initially increases and then it is reduced as the number of CBT cycles accumulates, while the residual elongation-to-fracture decreases monotonically. At CBT cycles close to the failure of the material (*i.e.* 10 and 12 CBT cycles, RD cases), the subsize tensile coupons fail almost as soon as they re-yield.

It should be noted that the results of Figure 7 do not contain the bending stress that is induced when the curved subsize tensile specimen is straightened before being loaded in tension. This bending stress is elastic, as could be verified easily by straightening the curved specimens by hand before installing them in the tensile testing machine. As a result of that, it is linearly distributed in the test section. Because of the existing bending stress, as well as the residual stresses left from the CBT pre-straining, instead of labeling the axes in Figure 7 as stress and strain, which would imply only the tensile stress and strain is being applied over the gage length, Force/Initial Area and Displacement/Gage Length ×100, were used. Furthermore, the results of Figure 7 do not include the residual stresses that will result from the CBT loading. Because of both of these reasons, it was not attempted to plot the interrupted test results as true stress-true strain, using the initial configuration as stress-free. Future work will couple a recently developed crystal plasticity model [16] for cyclic deformation with finite elements, to estimate the residual stresses after a number of CBT cycles. In a parallel effort, the cyclic plastic response of AA-6022-T4 will be assessed from tension-compression experiments [17] and a suitable non-linear kinematic hardening model of the Chaboche family (e.g., [18,19,20,21]) will be calibrated to be used in a finite element analysis of CBT.

The microstructural analysis of the material extracted during the wire-EDM processing of the subsize tensile specimens is described next.

## 4. Microstructural Characterization of Deformed Material

**Figure 8:**
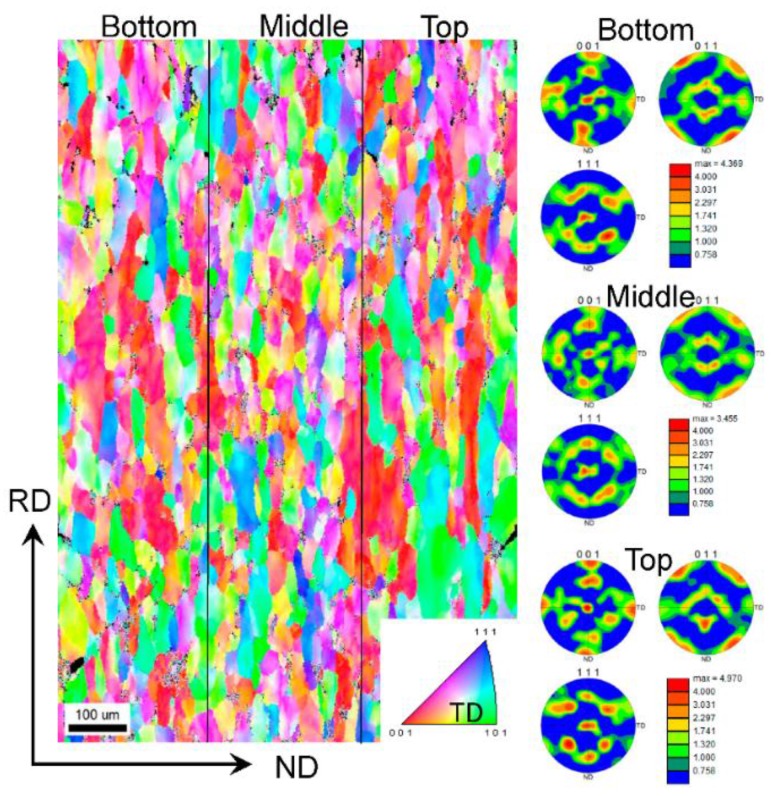
EBSD orientation map over entire thickness illustrating grain structure and pole figures showing crystallographic texture at three locations in a deformed sample after 12 cycles of CBT processing in the RD direction.

Figure 8 shows an EBSD map of a deformed sample after 12 cycles of CBT processing in the RD direction. The grains have significantly elongated in the RD and slightly contracted in the ND. Based on the grain structure, the deformation appears uniform throughout the sample. To further evaluate the uniformity of deformation, the through-thickness texture gradient was examined. Figure 8 also shows pole figures at top, middle, and bottom locations in the sheet. These pole figures confirm that the texture evolves uniformly through the thickness of the sheet. The particular texture component that develops is a <111> fiber, which is a known texture component forming under tension of FCC metals. For comparison, the texture evolution in a sample deformed in simple tension pulled to failure was examined.

**Figure 9:**
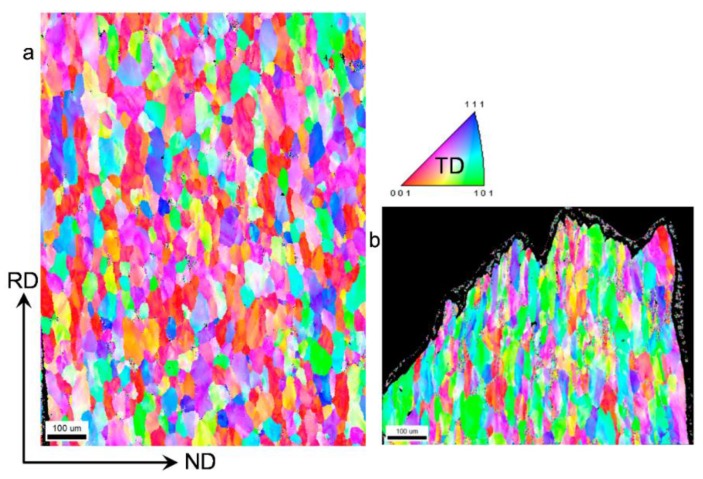
EBSD orientation maps over entire thickness of a specimen deformed to fracture in tension along the RD direction showing grain structure within: (**a**) gauge section of the specimen but outside necked region and (**b**) necked region.

Figure 9a,b shows the grain structure in a region away from the localized region within the gauge section and within the localized/failed region of the sample, respectively. While the grains away from the localized region show very little plastic deformation, the grains within the failure region are substantially elongated and appear similar to those in the CBT sample deformed to 12 cycles. However, in the latter case grains are similarly deformed throughout the sample to large strains because continuous bending/unbending deformation delays necking.

**Figure 10:**
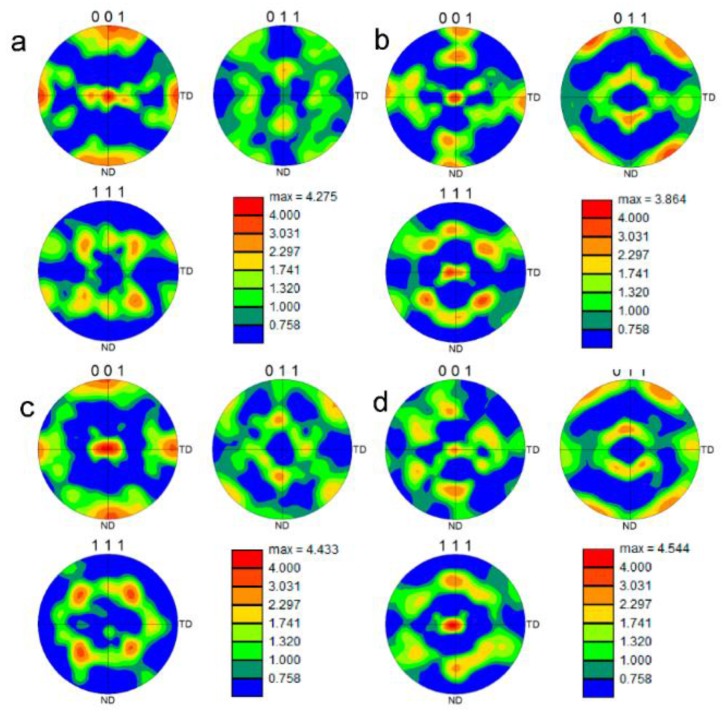
Pole figures showing a comparison of texture in (**a**) as received material, deformed materials after (**b**) 12 cycles of CBT along the RD direction, and simple tension along the RD direction (**c**) within gauge section of the specimen but outside necked region and (**d**) within necked region.

Figure 10 compares the texture evolution of the material deformed in CBT to 12 cycles and the material deformed in tension. The material away from the neck region deformed in tension (Figure 10c) does not show evidence of substantial texture evolution and the <111> fiber is not present. However, the material in the necked region (Figure 10d) underwent substantial texture evolution, which is similar to the texture developed in CBT to 12 cycles (Figure 10b). Unlike the sample deformed in simple tension, the CBT sample deformed uniformly throughout, preserving the integrity to very high strains and thus allowing texture and microstructure to substantially evolve.

**Figure 11:**
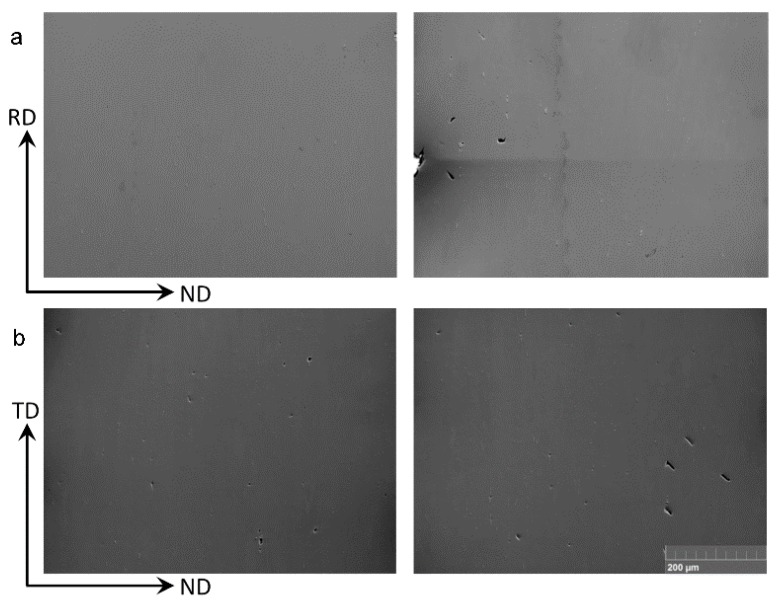
SEM images showing damage formation in terms of voids in the material after (**a**) 12 cycles of CBT along the RD direction and (**b**) 14 cycles of CBT along the TD direction. Two randomly selected locations along the sample length are shown for each sample over entire thickness.

Figure 11a shows SEM micrographs of the sample deformed in CBT to 12 cycles along the RD. The entire specimen thickness was imaged. We observe the evidence of ductile damage formation in terms of particle fragmentation and decohesion from the matrix as well as surface fracture, but no necking. Similar evidence is present in the sample deformed to 14 CBT cycles along the TD (Figure 11b).

**Figure 12:**
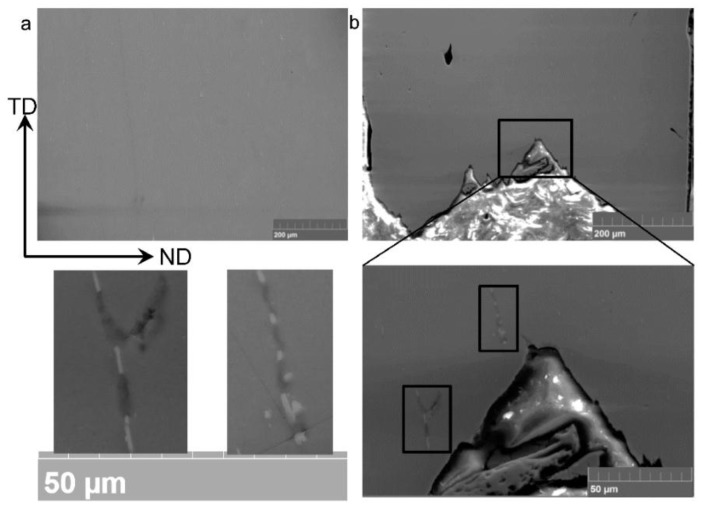
SEM images showing damage formation in terms of voids in the material after simple tension to failure (**a**) within gauge section of the specimen but outside necked region and (**b**) within necked region. Entire specimen thickness is imaged.

In contrast, Figure 12b shows that damage in terms of particle fragmenting develops locally under tension and quickly causes necking. No damage is found away from the necked region in tension (Figure 12a).

## 5. Conclusions and Outlook

In this work, a custom CBT machine suitable for Digital Image Correlation observations and for testing strips and sheets was designed and fabricated. Here, the machine is utilized for testing of AA-6022-T4 strips. The present investigation led to the following conclusions:

(a) The CBT process postpones the onset of necking. Experiments on 1 mm thick AA-6022-T4 showed increased elongation in CBT over conventional simple tension. Uniform depletion of ductility over the entire gauge length is possible using CBT.
(b) The CBT tests show decreasing axial load with increasing roller depth, which is significant for achieving innovative forming processes at reduced load capacity requirements.
(c) Subsize tensile samples extracted from the CBT specimens gave a higher yield stress and decreased elongation response relative to the simple tension response of the as-received material. The strength initially increases after CBT processing to a certain number of cycles, but then decreases with less elongation achieved for increasing numbers of CBT cycles.
(d) Microstructure characterization revealed that the material under CBT preserves higher integrity to large plastic strains than under simple tension. In the former case damage is distributed uniformly throughout the material while in the latter case damage evolves rapidly in a localized region leading to necking and fracture.
(e) The ability to delay ductile fracture during CBT processing to large plastic strains results in formation of a strong <111> fiber texture, which forms uniformly throughout the material. Therefore, the texture resulting from CBT deformation was similar to that around the neck in uniaxial tension.

In closing, note that there are other sheet metal forming processes that produce localized deformation, similar to the 3-point bending effect in CBT. For example in spin forming, a stationary tool contacts a spinning blank to create an axisymmetric component [22,23]. Alternatively, in incremental sheet metal forming (ISF) a hemispherical tool locally deforms the sheet. In both processes, only a small portion of the sheet is plastically deformed at each instant. Note that while there is more global, as opposed to localized, loading in CBT, the loading in the parts of the specimen outside of the rollers is elastic (see Figure 6), and thus does not contribute to the plastic deformation. Similarly to the CBT process, the strains achieved during ISF are well above what is possible in standard sheet forming. One of the hypotheses behind this enhanced necking limit [24,25] is the CBT effect as described in this paper.

Furthermore, the CBT phenomenon has been empirically observed in industry during deep drawing. The failure strains of a sheet which is pulled through a drawbead (*i.e.*, has been bent and unbent three times before entering the die cavity area) are considerably higher than those of the original sheet [26]. Reciprocating rollers or a wavy contour over which the material is traversed could be incorporated into a deep drawing process, either in the binder area or in the forming cavity, in order to achieve the increased elongation observed during CBT processing. These would produce lightweighting technologies for less ductile materials by capitalizing on the CBT effect.

## Figures and Tables

**Table 1:** Chemical composition of the AA-6022-T4 alloy (wt %).

Si	Fe	Cu	Mn	Mg	Cr	Zn	Ti	Tin	Al
0.90	0.10	0.045	0.053	0.57	0.027	0.016	0.025	<0.02	balance

**Table 2:** Interrupted continuous-bending-under-tension (CBT) experiments.

**Test Conditions**	Bending Depth	1.75 mm
	Cross-Head Velocity	0.86 mm/s
	Carriage (Aka Roller) Velocity	66 mm/s
**CBT Cycles Performed**	RD (Rolling Direction)	1, 4, 6, 8, 10, 12
	TD (Transverse Direction)	1, 4, 6, 8, 10
